# Molecular epidemiology of dengue viruses in southern China from 1978 to 2006

**DOI:** 10.1186/1743-422X-8-322

**Published:** 2011-06-26

**Authors:** Weili Wu, Zhijun Bai, Houqing Zhou, Zeng Tu, Meiyu Fang, Boheng Tang, Jinhua Liu, Licheng Liu, Jianwei Liu, Weijun Chen

**Affiliations:** 1 Key Laboratory of Genome Sciences and Information, Beijing Institute of Genomics, Chinese Academy of Sciences, Beijing, 100029, China; 2 Guangzhou Medical Research Institute, Yan-ling, Guangzhou, 510507, China; 3 Shenzhen Second People's Hospital, Shenzhen, 518035, China; 4 College of Biotechnology, Southwest University, Chongqing, 400715, China; 5 Beijing Genomics Institute in Shenzhen. Shenzhen, 518000, China

## Abstract

To investigate molecular epidemiology of dengue viruses (DENV) in southern China, a total of 14 dengue isolates were collected in southern China during each epidemic year between 1978 and 2006 and their full-length genome sequences were obtained by using RT-PCR method. The E gene sequences from additional 6 dengue fever patients in Guangzhou in 2006 were also obtained by using RT-PCR method. Combined with DENVs sequences published in GenBank, phylogenetic analysis and recombination analysis were performed. One hundred and twenty-five E gene sequences and 60 complete genome sequences published in the GenBank were also involved. Phylogenetic analysis showed that there was a wide genetic diversity of DENVs isolated in southern China. DENV-1 strains exist in almost all of the clades of genotype I and IV except the Asia 1 clade of genotype I; DENV-2 stains are grouped into four of the five genotypes except American genotype. DENV-4 strains are grouped into 2 genotypes (I and II). Phylogenetic analysis also showed that all DENV-4 isolates and two DENV-2 isolates were closely related to the prior isolates from neighboring Southeast Asia countries. The DENV-1 strain isolated during the 2006 epidemic is highly homologous to the strains isolated during the 2001 epidemic.

Recombination analysis showed no inter-serotype recombination, but 22 intra-serotype recombination events were found across the 32 complete genomes of all Chinese isolates. The study suggested that dengue fever epidemic in Southern China over the past 30 years presented two important modes, 1) imported-cases-induced endemic prevalence; 2) endogenous epidemic outbreak with natural epidemic focus. Recombination may play an important role in dengue virus evolution and adaptation.

## Background

Dengue fever (DF) and two more severe syndromes, dengue shock syndrome (DSS) and dengue hemorrhagic fever (DHF) are important mosquito-borne diseases in tropical and subtropical regions [1,2]. Since the first documented outbreak in 1779 in Jakarta, Indonesia, outbreaks have been documented in tropical and subtropical regions. It has been the maximum public health burden in South-East Asia countries [3]. Dengue epidemiology changes fast among epidemic countries, and keeps on expanding to the non-epidemic area [1]. Since the pathogens were first discovered by Japanese scientists in 1943, dengue viruses (DENV) were isolated from almost all South-East Asia countries including Thailand, Cambodia, Vietnam, Laos, Myanmar, Malaysia, Philippines, and Indonesia [3]. The dengue virus belongs to Flaviviridae family, and has four closely related but different serotypes (DENV-1 through -4) in nature that are circulating or co-circulating [1,2]. Their genetic diversity remains wide, due to 1) absence of a proof-reading capacity in RNA-dependent RNA polymerases [4], 2) emergence of different lineages or clades during epidemic [5,6], 3) increasing natural recombination [7-10], and 4) co-circulation of more than one serotypes in a locality [11-14].

Since imported DF epidemic had been reported in Hankou, Hangzhou, Shanghai and Guangzhou in 1920s and 1940s [15], there was no DF case reported in China till the outbreak occurred in 1978 in Foshan, Guangdong Province, with DENV-4 infection [16]. And then DF was prevalent in Guangdong, Guangxi and Hainan province. A DENV-1 epidemic occurred in 1979 in Zhongshan, Guangdong Province. This serotype of virus continued causing outbreaks over 2-3 years intervals. It become the dominating serotype and caused the latest outbreak in 2006 [17,18]. DENV-3 epidemic was only recorded once in 1980 at Zhan County, Hainan Island, where in 1985 DENV-2 caused an epidemic during which the first DHF case was reported [19]. After this DENV-2 epidemic, DENV-2 continued to be transmitted into Guangdong, Guangxi and Hainan until 2001, including three outbreaks in Foshan in 1993, 1998 and in Jiangmen in 2001 [20]. The second DENV-4 outbreak occurred in Guangzhou city in 1990 [20]. It was estimated more than 700,540 hospitalized cases with 513 deaths from 1978 to 2007 [15]. Although the first isolate was sampled 30 years ago, dengue epidemic in southern China keeps increasing [15]. Lack of longitudinal research on dengue epidemics has hampered our understanding of dengue molecular origin and evolution in China

In this study, we determined the complete genome sequences of 14 dengue isolates collected in southern China during each epidemic year between 1978 and 2006 and E gene sequences from six patients of Guangdong, 2006. In combination with those published sequences in GenBank, we conducted an extensive molecular epidemiological analysis, aiming to determine where the DENV isolates in China originally came from, and what shaped their evolution.

## Materials and methods

### Ethics statement

This research was approved by the Review Board of Guangzhou Medical Research Institute, the Review Board of Shenzhen Second people's Hospital, the Review Board of Beijing Institute of Genomics, the Review Board of Beijing Genomics Institute in Shenzhen and the Review Board of Southwest University. Written informed consent was obtained from the patient for publication of this case report and accompanying images. A copy of the written consent is available for review by the Editor-in-Chief of this journal.

### Sera

Six dengue fever patients' sera were collected in Guangzhou city during the epidemic of DENV-1 in 2006 by Guangzhou Medicine Institute. These sera were collected within 7 days after onset of symptoms and stored at -20°C. All sera were tested positive for DEN-1 IgM by indirect immunofluorescence assay at the Guangzhou Medicine Institute.

### Viruses

Fourteen DENV strains were obtained from patients sera in DF epidemic in China during 1978 to 2006 (Table 1). Sucking BALB/c mice and C6/36 cells were used to isolate viruses from clinical specimens. Serotypes of those isolates were confirmed by indirect immunofluorescence using anti-DENV monoclonal IgG (Guangzhou Medicine Institute, China). These isolates included eight DENV-1 isolates collected from Guangzhou and Chaozhou City of Guangdong province in 1991, 1995, 1997, 1999, 2003 and 2006 epidemic. Four DENV-2 isolates were collected from Fusan and Jiangmen City of Guangdong Province in 1993, 1998 and 2001 epidemic. Two DENV-4 isolates were collected in Foshan City during 1978 epidemic and in Guangzhou city during 1990 epidemic respectively.

**Table 1:** Description of DENV isolates sequenced in this study

Serotype	**Isolate **^***a***^	**Passage history (host, no. of passages)**^***b***^	Locality	Year
	GD03/91	SMB, 3; C6/36, 1	Guangzhou	1991
	GD95/95	SMB, 2; C6/36, 1	Guangzhou	1995
	GD01/97	SMB, 1; C6/36, 1	Chaozhou	1997
DENV-1	GD99/99	SMB, 1; C6/36, 1	Chaozhou	1999
	GD54/03	SMB, 1; C6/36, 1	Guangzhou	2003
	GD66/03	SMB, 1; C6/36, 1	Guangzhou	2003
	GD01/06	SMB, 1	Guangzhou	2006
	GD02/06	SMB, 1	Guangzhou	2006

	GD01/93	SMB, 2; C6/36, 1	Foshan	1993
DENV-2	GD06/93	SMB, 2; C6/36, 1	Foshan	1993
	GD08/98	SMB, 1; C6/36, 1	Foshan	1998
	GD01/01	SMB, 1; C6/36, 1	Jiangmen	2001

DENV-4	GD07/78	SMB, 2	Foshan	1978
	GD09/90	SMB, 2; C6/36, 1	Guangzhou	1990

^*a*^All isolates were isolated from patients with primary infection manifesting classic DF symptoms at Guangdong Province, People's Republic of China.

^*b*^SMB, sucking mouse brain; C6/36, *Aedes albopictus *cell line.

### Viral RNA extraction and RT-PCR methods

Viral RNA was extracted using TRIzol reagent (Invitrogen Corp., California, USA) according to the manufacturer's instruction. The cDNA was synthesized by reverse transcription from 10 μL of RNA at 42°C for 50 min in a 50 μL solution containing 50 mM Tris-HCl (pH 8.3), 75 mM KCl, 3 mM MgCl2, 10 mM DTT, 100 ng of the random hexamer primers, 200 U of Superscript II reverse transcriptase (Invitrogen), 25 U of RNasin (Invitrogen) and 0.5 mM dNTPs. The PCR was performed in a 25 μl mixture containing 2 μl of cDNA, 10 mM Tris/HCl (pH 8.4), 50 mM KCl, 2.5 mM MgCl2, 100 μM each dNTP, 1 U Taq DNA Polymerase (Shanghai Promage Corp., Shanghai, China), 0.25 μM each primer (Additional file 1, Table S1-S3). The amplification reactions consisted of an initial denature step of 3 min at 95°C, followed by 35 cycles of 30 s at 94°C, 30 s at 55°C, 60 s at 72°C.

### Viral genome sequencing and E gene sequencing

The complete genomes of the viruses were sequenced by a PCR-by-PCR strategy. At the same time, Envelope (E) gene from six patients' sera was also amplified. All primers were synthesized by BGI (Beijing Genomics Institute, Beijing, China) according to the reference sequence data of DENV-1 Singapore strain S275/90 [GenBank: M87512], DENV-2 Jamaica strain 1409/88 [GenBank: M20558], and DENV-4 Dominica strain 814669/81 [GenBank: AF326573]. Amplified products were detected by agarose gel electrophoresis and sequenced using ABI 3730xl DNA Analyzers (Applied Biosystems, Foster City, CA).

### Assembly of Genome Sequences and Sequence Analysis

Genome assembly was performed independently by distinct operators using Phred-Phrap-Consed [21]. The consensus sequence was yielded exactly with BLASTN and custom script which was written by Perl for all strains. A single contig was obtained for each of the 14 isolates. The single contig was aligned to reference by BLASTN with the E-value of 1e-5. The computation of substitution tables were performed using a custom program written by Perl.

Neighbor-joining trees were constructed using MEGA version 3.1 with the Kimura-2 parameter corrections of multiple substitutions [22]. Reliability of nodes was assessed by bootstrap resampling with 1,000 replicates. One hundred and twenty-five E gene sequences and 60 complete genome sequences published in the GenBank were also involved.

RDP3 was used for recombination analysis. We employed Bootscan, Chimera, GENECONV, MaxChi, and RDP methods incorporated in RDP3 Beta 27 program [23-27]. General recombination settings for all methods were as follows: Sequences were considered as linear, and the highest acceptable P-value was set to 0.01, and event detected by two or more methods was taken into consideration.

## Results

### Nucleotide sequence accession numbers

The determined DENV nucleotide sequences were deposited in GenBank database under the accession numbers FJ196841-FJ196860.

### Phylogenetic tree of DENV-1, -2, and -4 generated by E gene sequences

**Figure 1:**
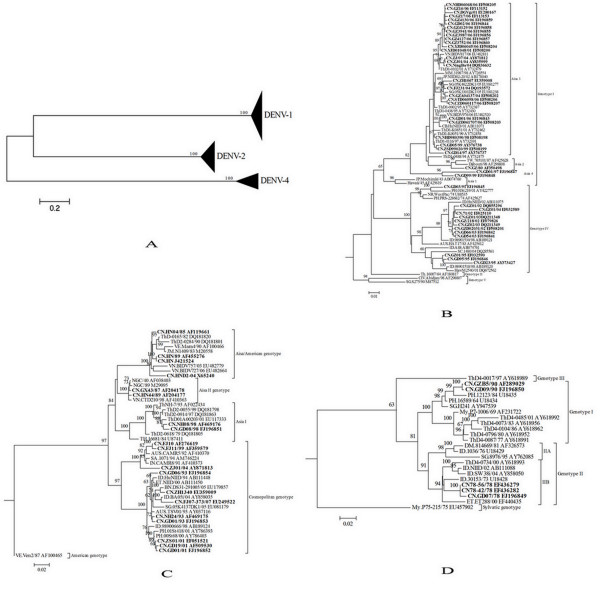
**Neighbor-joining phylogenetic trees of 145 E gene sequences were conducted using Mega3.1 with the Kimura-2 parameter corrections of multiple substitutions**. Bootstrap values (in percentage >65%) on each node were generated by using 1000 replications. The branch lengths are proportional to the number of nucleotide changes. All the Chinese isolates are in bold for clarity. A: phylogenetic tree of serotype DENV-1, DENV-2 and DENV-4 virus; B: phylogenetic tree of the DENV-1 isolates; C: phylogenetic tree of the DENV-2 isolates; D: phylogenetic tree of the DENV-4 isolates.

To determine the DENV molecular epidemiology in southern China, 145 E gene sequences were used to infer a ML phylogenetic tree (Figure 1), including 1) 20 E gene sequences determined in the study, 2) 47 sequences with China localities available from GenBank, and 3) 78 representative sequences from GenBank referring to diverse genotype and geographic localities.

The DENV-1 isolates from Guangdong belong to genotype I and IV (Figure 1B). Within genotype I and IV, several sub-genotypes can be identified. Two isolates (CN.GD01/97 and CN.GD99/99) determined in the study clustered together as a single clade with a 96% bootstrapping support, which was named as a new Asia-4 clade. DENV-1 strains isolated in 2001 and 2006 belong to Asia 3 clade in genotype I. However, the stains of 2006 could further be divided into three different sub-clades. Eleven strains of 2006 positioned at the end of the Asia 3 clade and the other four strains positioned at two different sub-clades. Even the eight strains (two isolates and six patients' sera) collected in Guangzhou city during the epidemic of DENV-1 in 2006 also clustered into 2 subclades in Asia 3 clade. All eight strains collected during 2002 and 2003 period at Guangzhou were identical and clustered closely as genotype IV type.

Chinese DENV-2 isolates were characterized into four genotypes except for American genotype (Figure 1C). Broadly, those collected in pre-1990s fell into Asia/American and Asia II genotypes, while the post-1990s belonged to Asia I and Cosmopolitan genotypes. Two isolates collected in 1998 in Foshan, which belonged to Asia I genotype, were identical to the strain from Thailand in 1993. Three isolates of Jiangmen in 2001 epidemic showed closely identical to the strain from Philippines in 2000 epidemic.

Two DENV-4 epidemics occurred in China in the year of 1978 and 1990. Five isolates collected from the two epidemic years belonged to genotype I and genotype II (Figure 1D). Two isolates collected during 1990 epidemic belonged to genotype I and clustered closely with a strain isolated in 1984 epidemic of Philippines (PH. 12123/84) [GenBank: U18435]. The three other DENV-4 isolates from 1978 epidemic clustered together and fell into genotype II. They resided in phylogenetic tree much closer to the Indonesia isolate 30153 (ID. 30153/73) [GenBank: U18428] collected in 1973 when there was a severe dengue epidemic.

### Phylogenetic tree of DENV-1, -2, and -4 generated by ORF sequences

**Figure 2:**
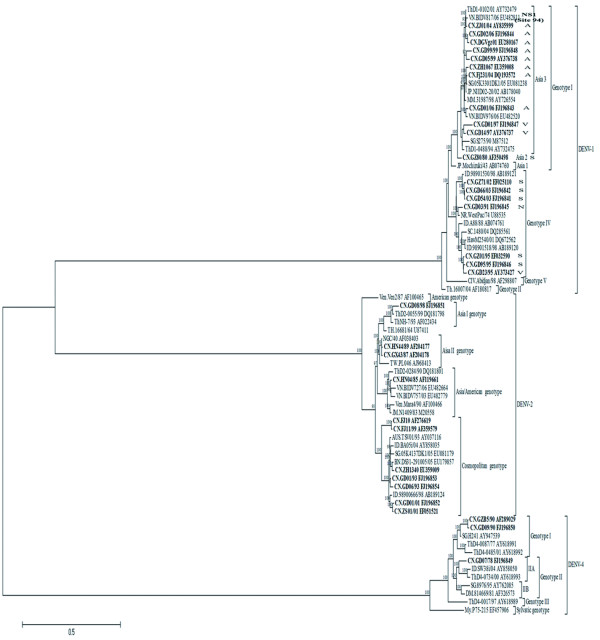
**Neighbor-joining phylogenetic trees of 74 complete coding regions were conducted using Mega3.1 with the Kimura-2 parameter corrections of multiple substitutions**. Bootstrap values (in percentage >65%) on each node were generated by using 1000 replications. The branch lengths are proportional to the number of nucleotide changes. All the Chinese isolates are in bold for clarity.

Based on the 74 complete coding regions (14 determined in the study and 60 from GenBank), we constructed maximum likelihood (ML) phylogenetic tree (Figure 2).

In the study, the whole genome and the single E gene phylogenetic tree are in good coincidence with only little differences. Two DENV-1 isolates, CN.GD01/97 and CN.GD99/99, were assumed to be new members of a defined Asia-4 clade in E gene tree (Figure 1B), while in the coding region phylogenetic tree; they clustered into the Asia-3 clade (Figure 2).

### Recombination detection

**Figure 3:**
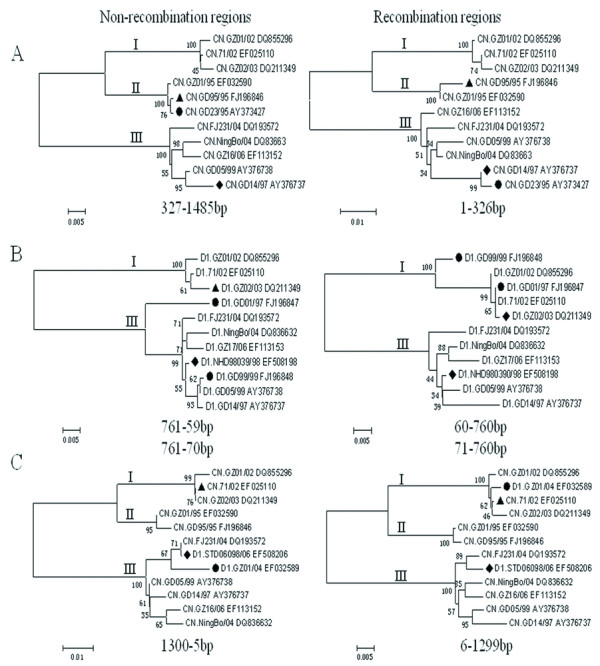
**Phylogenetic analysis based on non-recombination regions (Left) and recombination regions (Right) of event 1 (A), 2 (B), and 3 (C) constructed by Mega 3.1 using Neighbor-joining methods**. The event numbers are in accordance with table 2. Black circle, square, and triangle indicate the sequences of recombinant, major parent and minor parent, respectively. The trees are mid-rooted for clarity.

RDP3 software was employed to examine the potential recombination events of all the Chinese isolates (43 DENV-1,20 DENV-2,2 DENV-3,5 DENV-4) on E gene. Three recombinant events were detected on the E gene of DENV-1 with low P-value (P < 0.01) (Table 2), which was confirmed by phylogenetic construction (Figure 3). Neither DENV-2 nor DENV-4 has any evidence of such recombination across their counterparts. The potential recombination events were also analyzed across the 32 complete ORF of all the Chinese isolates by RDP3 software. No inter-serotype recombination was detected, but 22 intra-serotype potential recombination events (17 for DENV-1, 5 for DENV-2) were confirmed by more than two methods (Table 3).

**Table 2:** Detected recombination events on the E gene region of Chinese DENV-1*

Event	Daughter	Major Parent	Minor Parent	**Region**^**†**^
1	D1.GD23/95 (AY373427)	D1.GD14/97 (AY376737)	D1.GD95/95 (FJ196844)	1-326

2	D1.GD01/97 (FJ196847)	D1.NHD98039/98 (EF508198)	D1.GZ02/03 (DQ211349)	60-760
	D1.GD99/99 (FJ196848)			71-760

3	D1.GZ01/04 (EF032589)	D1.STD06098 (EF508207)	D1.71/02 (EF025110)	6-1299

*Recombinant region and parental sequences were identified by five procedures (Bootscan, Chimarea, GENECONV, MaxChi, and RDP) integrated in RDP3 Package with a multiple comparison corrected P-value < 0.01.

^†^Region coordinates are nucleotide positions of detected breakpoints in recombinant E gene sequence (1485bp).

**Table 3:** Recombination events detected by RDP3^†^

Recombinant Event Number	Breakpoint Positions in Recombinant Sequence		Recombinant Sequence(s)	Minor Parental Sequence	Major Parental Sequence
				
	Begin	End			
1	677	10104*	D2.GD06/93	D2.GD01/93	D2.GD08/98
2	5514	6548*	D1.GD05/99	D1.GD95/95	D1.GD01/06
3	1*	7487	D1.GD01/97	D1.GD14/97	D1.GD95/95
			D1.GD99/99	D1.ZJ01/04	D1.GZ01/95
4	900	1605	D1.GD01/97	D1.GZ71/02	D1.GD14/97
			D1.GD99/99	D1.GZ01/95	D1.ZJ01/04
5	5763	6401	D1.GD99/99	D1.GD66/03	D1.GD01/06
6	1*	466	D1.GD05/99	D1.GD23/95	D1.GD99/99
7	5585	6219	D1.GD01/97	D1.GD66/03	D1.GD14/97
8	749	1167	D1.GD23/95	D1.GD14/97	D1.GD95/95
9	8240	9343	D2.GD08/98	D2.GD01/93	D2.GZ44/89
10	5457	6201*	D1.GZ71/02	D1.GZ01/95	D1.GD54/03
11	1606*	2555	D1.GD14/97	D1.GD99/99	D1.GD01/97
12	3711	4913	D2.GD01/01	D2.GD06/93	D2.ZS01/01
13	2588*	2898	D1.GD23/95	D1.GD14/97	D1.GZ01/95
14	5171	5506	D1.GD23/95	D1.GD14/97	D1.GD95/95
15	7890	8795	D1.GD01/97	D1.GD99/99	Unknown
16	2708	3121	D1.GD01/06	D1.GD54/03	D1.ZJ01/04
17	4889	5368	D2.GD08/98	D2.ZS01/01	D2.GZ44/89
18	9645	10146*	D1.GD95/95	D1.GD03/91	D1.GZ01/95
19	7890*	8385	D1.GD99/99	D1.GZ71/02	Unknown
20	8820	9106	D1.GD99/99	D1.GD23/95	D1.GD05/99
21	2335	2398*	D1.GD95/95	Unknown	D1.GD23/95
22	1043*	10104*	D2.GD06/93	D2.GD01/93	D2.ZS01/01

^† ^= 22 final recombination events resulting from at least two methods confirmation integrated in RDP3 package with a multiple comparison corrected P-value < 0.01 and one-by-one manual checking. Only the closest major and minor parental sequences were shown in the table. Recombinants of DENV-1 were bolded for clarity.

* = The actual breakpoint position is undetermined (it was most likely overprinted by a subsequent recombination event).

Minor Parent = Parent contributing the smaller fraction of sequence.

Major Parent = Parent contributing the larger fraction of sequence.

Unknown = Only one parent and a recombinant need be in the alignment for a recombination event to be detectable.

## Discussion

Since the first well documented DF outbreak in Foshan of Guangdong province in 1978, dengue has been reported periodically in southern China, with the latest epidemic at Guangdong province in 2006. In this study, 14 Dengue isolates from 1978 to 2006 were sequenced and analyzed for molecular evolution.

Dengue virus exists as four antigenically distinct viruses designated as serotypes (DENV-1 through DENV-4), belonging to genus *Flavivirus *of family *Flaviviridae*. It has a positive-sense RNA genome that is translated as a single polyprotein and posttranslationally cleaved into three structural proteins and seven nonstructural proteins [28]. The envelope protein (E) is considered to be the immunodominant protein [29]. Dengue viruses also could be divided into different genotypes by the E gene [30,31]. So there is correlation between serotypes and genotypes of Dengue virus. Phylogenetic trees of DENV-1, -2 and -4 generated by ORF and E gene sequences showed that there was a wide genetic diversity of DENVs isolated in southern China (Figure 1, Figure 2). DENV-1 strains exist in almost all of the clades of genotype I and IV but Asia 1 clade of genotype I (Figure 1B, Figure 2). DENV-2 strains are grouped into four genotypes except American genotype (Figure 1C, Figure 2). DENV-4 strains are grouped into 2 genotypes (I and II) (Figure 1D, Figure 2). It also showed that different serotypes and genotypes epidemic prevalence exist in the same city and even during the same epidemic (Figure 1, Figure 2). It indicated that these dengue viruses maybe have different origination.

Some of isolates, especially the first emerging of certain dengue serotype strains in southern China were closely related to those strains which were isolated in prior epidemics from neighboring Southeast Asia countries. It indicated that these dengue epidemics may be imported into China from the neighbor countries. For DENV-2, there were two outbreaks in Foshan in 1993 and 1998. Isolates from 1998 epidemic was closely related to the isolates from 1993 epidemic in Thailand (Figure 1C). The same situation was showed in DENV-4 isolates from 1978 and 1990 epidemics. Isolates from 1978 epidemic was closely related to the isolates from 1973 epidemic in Indonesia (Figure 1D). Isolates form 1990 epidemic was closely related to the isolates from 1984 epidemic in Philippines (Figure 1D). It indicated that these epidemics maybe imported into China from neighbor countries.

Although some data showed the dengue fever were imported from neighbor countries, those epidemics with the same serotype of DENV during continues years showed that endemic infection of dengue circulating locally may be also the important cause of Dengue epidemic in southern China. In the study, two isolates (CN.GD01/97 and CN.GD99/99) clustered together as a new Asia-4 clade (Figure 1B), suggesting that the isolates of 1999 maybe originated from the isolates of 1997. Sequence analysis showed that the DENV-1 strains isolated during the 2003 epidemic were closely related to the strains isolated during the 2002 epidemic of Guangzhou, suggesting that the 2002 strains had evolved in local and eventually cause the epidemic of 2003 (Figure 1B). Similar case was also showed in Guangzhou outbreak of DF in 2006. The DENV-1 strain isolated during the 2006 epidemic is closely related to the strains isolated during the 2001 epidemic (Figure 1B). It suggested that DENV probably circulated in local and caused the epidemics of 2001 and 2006. Moreover, there were several clear divisions within the Chinese 2006 isolates in the tip of the tree, even the eight strains collected in Guangzhou city by us during the epidemic of DENV-1 in 2006 also clustered into 2 subclades in Asia 3 clade, showing viral evolution or increase of genetic complexity even during a single epidemic (Figure 1B).

Recombination plays a role in dengue virus evolution and adaptation [7]. We analyzed the potential recombination events of all the Chinese isolates (43 DENV-1,20 DENV-2,2 DENV-3,5 DENV-4) on E gene. Three recombinant events were detected on the E gene of DENV-1 with low P-value (P < 0.01) (Table 2). The incongruence of phylogenetic trees constructed separately by recombinant regions and non-recombinant regions confirmed the recombinant events (Figure 3). The recombination event 1 showed recombination between present GD14/97 isolate [GenBank: AY376737] as the major parent and the GD95/95 isolate [GenBank: FJ196846] as the minor parent, which led to a recombinant isolate of GD23/95 [GenBank: AY373427](Figure 3A). The recombination event 2 showed recombination between present NHD98039/98 isolate [GenBank: EF508198] as the major parent and the GZ02/03 isolate [GenBank: DQ211349] as the minor parent, which led to two recombinant isolates of GD01/97 [GenBank: FJ196847] and GD99/99 [GenBank: FJ196848](Figure 3B). The recombination event 3 showed recombination between present STD06098/06 isolate [GenBank: EF508206] as the major parent and the 71/02 isolate [GenBank: EF025110] as the minor parent, which led to a recombinant isolate of GZ01/04 [GenBank: EF032589](Figure 3C). These studies of recombination provided an important context to understand the evolution of these viruses and sequence diversity generated in viruses in China.

We also analyzed the potential recombination across the 32 complete ORF of all the Chinese isolates, and found no inter-serotype recombination occurred. The results are coincided with previous data [8,10,32]. However, we found 22 intra-serotype potential recombination events (17 for DENV-1, 5 for DENV-2) (Table 3). Interestingly, for DENV-1, 13 of 17 potential recombination events happened in the strains isolated in 1995, 1997 and 1999 epidemics (Table 3), prior to which, in 1995, the severest dengue outbreak ever reported, indicating a possibility of viruses escaping human immunity by recombination following epidemic. However, whether the presumption makes sense or just a coincidence remains an open question.

## Conclusions

Our study suggested that Dengue fever epidemic in Southern China over the past 30 years presents two important modes, 1) imported-cases-induced endemic prevalence; 2) endogenous epidemic outbreak with natural epidemic focuses. Recombination analysis showed that recombination plays an important role in dengue virus evolution and adaptation.

## Conflict of interests declaration

The authors declare that they have no competing interests.

## Authors' contributions

WLW, HQZ, ZT and LCL carried out the genes sequencing and phlogenetics analysis. ZJB and MYF carried out the virus's culture and serotype identification. BHT and JHL participated in the samples detection. JWL and WJC designed the study, perform the data analysis and wrote the manuscript. All authors read and approved the final manuscript.

## Supplementary Material

Additional file 1 **Table S1-S3**. Overlapping PCR primers for DENV-1, DENV-2 and DENV-4 genome sequences Click here for file
