# Supplementary material for: Molecular epidemiology of dengue viruses in southern China from 1978 to 2006
Source: Virol J. 2011 Jun 26;8:322. doi: 10.1186/1743-422X-8-322 (PMC3138434; doi:10.1186/1743-422X-8-322)
Supplement: Additional file 1 — Table S1-S3. Overlapping PCR primers for DENV-1, DENV-2 and DENV-4 genome sequences [file 1743-422X-8-322-S1.DOC]

| Primers code | Forward primer | start | Reverse Primer | start |
| --- | --- | --- | --- | --- |
| DV.1_1 | ACAACCAACGGAAAAAGAC | 99 | CCTCTTTCCTGCTTGCTAAC | 495 |
| DV.1_2 | ATGAACAGGAGGAAAAGAT | 377 | GTGTCTCAGAGCCCAGGT | 826 |
| DV.1_3 | AACACCGACGAGACAAAC | 690 | TTGTGACCTCCGTCTTCA | 1088 |
| DV.1_4 | AGGAGCTACGTGGGTGGA | 982 | GCAGTTGTTCCATGTTCTG | 1419 |
| DV.1_5 | CGTGTGCTAAGTTTAAGTGT | 1278 | TGTGCATTGCTCCTTCTT | 1718 |
| DV.1_6 | TACCACTGCCTTGGACCT | 1581 | GACTGGTTTTTCTTTGTCAG | 2029 |
| DV.1_7 | AGACCCAGCATGGAACTG | 1875 | CAGCAGAATCCCTATTCCTA | 2323 |
| DV.1_8 | CATGGGACTTCGGTTCTA | 2190 | TTCCACACACACCCTCCT | 2588 |
| DV.1_9 | ACCAATGAAGTCCACACC | 2483 | CCATATGTTTGTCGTGAA | 2923 |
| DV.1_10 | CCAAAATCATAGGAGCAGAT | 2781 | GCCCTGCTGTTTGTGTGA | 3218 |
| DV.1_11 | GCCAAAATCCCACACTCT | 3094 | TGTCCACTTCTCCTGACC | 3494 |
| DV.1_12 | CGTTTCAAAGGAGAAGAC | 3383 | TCTACAGATGCCACCAGAC | 3819 |
| DV.1_13 | GGGGATGGGAACAACGTA | 3679 | CCCCAGATTTTGTTTTCTG | 4122 |
| DV.1_14 | TTCCCTTTATGCCTGTCC | 4007 | TCCTTTATCTTCATGGTTCC | 4392 |
| DV.1_15 | CCGATTTATCACTGGAGA | 4275 | AACTTGGTTCCAGTCTCTT | 4724 |
| DV.1_16 | GGGCAGGTCTCAAGTAGG | 4612 | ATGGCACTGACGTAGGTA | 5013 |
| DV.1_17 | GCGAAGTTGGAGCCATAG | 4881 | GACGCATAGTGAAAGTGG | 5327 |
| DV.1_18 | TCTTAGCTCCCACAAGAGTT | 5181 | TGATTTGATGCTTGGAACA | 5623 |
| DV.1_19 | GCAATGCAGTTATCCAAGA | 5505 | TCCAATTCTTCCTCTCCTC | 5908 |
| DV.1_20 | GAGCCGACAGGGTAATAGA | 5775 | CCATCAAAGCACCACCTT | 6219 |
| DV.1_21 | GCAATAGACGGGGAATACA | 6074 | GTTCTTCCATGGCGTGTC | 6518 |
| DV.1_22 | AGGGAAACTTCCACAACA | 6406 | AGACCTATCACCACGTATGC | 6798 |
| DV.1_23 | GGAGTTCTTTCTGATGGTGT | 6709 | AGGCGAGAAGTGGAACTC | 7115 |
| DV.1_24 | GAAAACACAACGGCAAAT | 6998 | TCCAGTGGCTAGTGTGATG | 7426 |
| DV.1_25 | CGTTGCAATAGATTTGGAC | 7279 | CGTTTCTCCTCTTTTTAACC | 7723 |
| DV.1_26 | AGCCCAAGGGGAAACACT | 7582 | GGACTCACCAATATCACACA | 8023 |
| DV.1_27 | GGATACACGAAAGGAGGA | 7877 | ATGTTGGCTTCCTGTGAG | 8324 |
| DV.1_28 | GGAATGCTAGTGCGAAATC | 8177 | CAAAGGGTGTGGTGTCAG | 8618 |
| DV.1_29 | ATGAGGTCAAGCCATCAG | 8496 | TGAAGCTCCCTCTCTCTGT | 8895 |
| DV.1_30 | GCAGTGTTCGTTGATGAAA | 8801 | CCTCTGTTATTCTTGTGTCC | 9203 |
| DV.1_31 | CAGTGGAGTGGAAGGAGA | 9082 | TCTCGGTGCCATGTTTTT | 9530 |
| DV.1_32 | CATGGAGGCCCAACTAAT | 9412 | GCGCCTTGTGATACTCTG | 9801 |
| DV.1_33 | GGCAACAAGTGCCTTTCT | 9678 | AGGGAACCACACCATTGA | 10119 |
| DV.1_34 | TGGAATAGGGTTTGGATAGA | 10007 | CGGCCTGACTTCATTTTA | 10406 |
| DV.1_35 | AAGGGGCACTCTGGTAAG | 10257 | GTCTCTCCCAGCGTCAAT | 10653 |

Table S1. Overlapping PCR primers for DENV-1 genome sequence

Table S2. Overlapping PCR primers for DENV-2 genome sequence

Table S3. Overlapping PCR primers for DENV-4 genome sequence

| Primers code | Forward primer | Start | Reverse Primer | start |
| --- | --- | --- | --- | --- |
| Den4_1 | GTGGACCGACAAGGACAGT | 15 | TTCGGTATTGACCAGTAGGG | 621 |
| Den4_2 | GACAACAGAAGGAATCAATAAGTG | 518 | CGTTGCCCCACCCTCTATCT | 1248 |
| Den4_3 | CATAACCACGGCAACAAGAT | 1139 | GCACTGTCTCCTACACCTATTACTAT | 2095 |
| Den4_4 | AAAAGTCAAGTATGAGGGTGCT | 1904 | TGCCAAATCCATAGTCTTCC | 2907 |
| Den4_5 | CGCACCTCCAGTGAATGA | 2735 | GAAGGTCAAGGAAAGGGCTA | 3938 |
| Den4_6 | TGGTAACACATTTTGACAACACT | 3883 | TCTTCCTGTGTGTTCTGATTTC | 5285 |
| Den4_7 | AGGCTGCGAACCCTGAT | 5116 | CTTCCACTGGCAAACTCC | 6373 |
| Den4_8 | TTTCAAGGAGTTTGCCAGTG | 6350 | TCCTGTGGTCCCAGTTCC | 7580 |
| Den4_9 | CTGGAACACGACCATAGCC | 7454 | GTTGGCTGTCGTGGTCATA | 8699 |
| Den4_10 | TGACCCAGTTGGCTATGACA | 8575 | TGGCTCTCCCTATCAGTTCATCC | 9781 |

| Primers code | Forward primer | start | Reverse Primer | start |
| --- | --- | --- | --- | --- |
| Den2_1 | AGTTGTTAGTCTACGTGCACC | 1 | TGTCTTCACACAGTTCACCAA | 580 |
| Den2_2 | ACGCAACGGAGAACCAC | 457 | TGTTGGGCAGCGAGATG | 1164 |
| Den2_3 | ACATCCAGGCTTCACCAT | 825 | TGGTAACGGCAGGTCTAA | 1593 |
| Den2_4 | GGCACTGTCACGATGGAA | 1471 | GTGAGGTGCTGCGTGAAT | 2359 |
| Den2_5 | AAGACAGCCCAGTCAACATA | 2108 | TTGTGTATGATAGCCTGGTCT | 3210 |
| Den2_6 | TGGAAGATTGAGAAAGCCTC | 3049 | ATTTGGGACGCACAAGATAG | 3948 |
| Den2_7 | TCAGAGCACCATACCAGAGAC | 3813 | GACGACACCGTTGCCATA | 4986 |
| Den2_8 | CGGTGTCGTCACAAGGAG | 4977 | CTTCAGGCGTGTTGATGTTA | 6028 |
| Den2_9 | AGCACAAAGAAGAGGGAGAGT | 5883 | CATCCGATAGCGAGAAGG | 7118 |
| Den2_10 | TCGCTATCGGATGCTATTC | 7106 | GTCTGGCTCGTATGTGGC | 8334 |
| Den2_11 | GCCACATACGAGCCAGAC | 8317 | AGACTCCTTCTCCCTCCATC | 9484 |
| Den2_12 | AGACAGATGGAGGGAGAA | 9430 | ATTGTTGCTGCGATTTGT | 10540 |
